# *TALE* Transcription Factors in Sweet Orange (*Citrus sinensis*): Genome-Wide Identification, Characterization, and Expression in Response to Biotic and Abiotic Stresses

**DOI:** 10.3389/fpls.2021.814252

**Published:** 2022-01-20

**Authors:** Weiye Peng, Yang Yang, Jing Xu, Erping Peng, Suming Dai, Liangying Dai, Yunsheng Wang, Tuyong Yi, Bing Wang, Dazhi Li, Na Song

**Affiliations:** ^1^College of Plant Protection, Hunan Agricultural University, Changsha, China; ^2^Hunan Provincial Key Laboratory for Biology and Control of Plant Diseases and Insect Pests, Hunan Agricultural University, Changsha, China; ^3^Horticulture College, Hunan Agricultural University, Changsha, China; ^4^National Center for Citrus Improvement Changsha, Changsha, China

**Keywords:** *Citrus sinensis*, genome-wide characterization, expression analysis, *TALE* transcription factor, biotic and abiotic stresses

## Abstract

Three-amino-acid-loop-extension (TALE) transcription factors comprise one of the largest gene families in plants, in which they contribute to regulation of a wide variety of biological processes, including plant growth and development, as well as governing stress responses. Although sweet orange (*Citrus sinensis*) is among the most commercially important fruit crops cultivated worldwide, there have been relatively few functional studies on *TALE* genes in this species. In this study, we investigated 18 *CsTALE* gene family members with respect to their phylogeny, physicochemical properties, conserved motif/domain sequences, gene structures, chromosomal location, *cis*-acting regulatory elements, and protein–protein interactions (PPIs). These *CsTALE* genes were classified into two subfamilies based on sequence homology and phylogenetic analyses, and the classification was equally strongly supported by the highly conserved gene structures and motif/domain compositions. *CsTALEs* were found to be unevenly distributed on the chromosomes, and duplication analysis revealed that segmental duplication and purifying selection have been major driving force in the evolution of these genes. Expression profile analysis indicated that *CsTALE* genes exhibit a discernible spatial expression pattern in different tissues and differing expression patterns in response to different biotic/abiotic stresses. Of the 18 *CsTALE* genes examined, 10 were found to be responsive to high temperature, four to low temperature, eight to salt, and four to wounding. Moreover, the expression of *CsTALE3/8/12/16* was induced in response to infection with the fungal pathogen *Diaporthe citri* and bacterial pathogen *Candidatus* Liberibacter asiaticus, whereas the expression of *CsTALE15/17* was strongly suppressed. The transcriptional activity of *Cs*TALE proteins was also verified in yeast, with yeast two-hybrid assays indicating that CsTALE3/CsTALE8, CsTALE3/CsTALE11, CsTALE10/CsTALE12, CsTALE14/CsTALE8, CsTALE14/CsTALE11 can form respective heterodimers. The findings of this study could lay the foundations for elucidating the biological functions of the *TALE* family genes in sweet orange and contribute to the breeding of stress-tolerant plants.

## Introduction

In plants, numerous transcription factors (TFs) have been identified and shown to play significant roles in the regulation of developmental processes, stress responses, and genetic control (Liu X. et al., 2019). TFs in the three-amino-loop-extension (TALE) gene family have established to be relatively numerous and highly conserved in different plant species (Choe et al., 2014). These genes are classified into two subfamilies, namely, the KNOX (KNOTTED-like homeodomain) and BEL (BEL1-Like homeodomain) subfamilies, which normally function as heterodimeric TF complexes that contribute to modifying physiological and biochemical properties, particularly those associated with the metabolism and biosynthesis of lignin (Yoon et al., 2014, 2017). TALE proteins have a distinctive common characteristic in that interactions can occur either between TALE and non-TALE members or among different TALE family members (Hudry et al., 2014). In barley, for example, BKN3 (KNOX protein) has been shown to interact with JUBEL1 and JUBEL2 (BEL proteins; Müller et al., 2001), whereas SHOOT MERISTEMLESS (STM), a MEINOX domain protein, has been demonstrated to be a common interacting partner of three BEL homeodomain members (ATH1, BLH3, and BLH9; Cole et al., 2006).

The nutritional and economic value of fruits is dependent to a large extent on their developmental status, which is often determined by *TALE* genes. In tomato (*Solanum lycopersicum*), for example, the *TALE* gene *TKN2/4* has been demonstrated to specifically influence fruit chloroplast development and thereby nutrient composition and flavor (Nadakuduti et al., 2014). Similarly, *LeT6/TKn2* has been reported to be involved in tomato fruit morphological development (Avivi et al., 2000). In addition, CcBLH6 had been found to play an active role in the lignification and lignin biosynthesis pathway of *Camellia chekiangoleosa* fruit (Yan et al., 2021). Moreover, the activity of *TALE* family members is believed have a considerable influence on the size, yield, and quality of fruit in many fruit crops, including *Actinidia chinensis*, *Fragaria vesca*, and *Litchi chinensis* (Shahan et al., 2019; Zhao et al., 2020; Brian et al., 2021).

In recent years, an increasing amount of evidence has accumulated to indicate that *TALE* genes play important roles not only in growth and development but also in the adaptation of stress responses in different plant species (Butenko and Simon, 2015). For instance, *GmSBH1*, the first *TALE* gene identified in *Glycine max*, has been shown to influence leaf phenotype and enhance plant tolerance to high temperatures or humidity (Shu et al., 2015). In *Populus*, the type I *KNOX* gene *PagKNAT2/6b* has been demonstrated to directly suppress gibberellin biosynthesis, thereby promoting phenotypic alteration and enhancing plant drought stress tolerance (Song X. et al., 2021). Similarly, POTH15, a type I KNOX gene, has been characterized as a regulator of photoperiodic development, meristem maintenance, and leaf development, and is believed to be involved in responses to plant hormone signal transduction and biotic or abiotic stresses, based on RNA sequencing and quantitative real-time PCR (qRT-PCR) validation (Mahajan et al., 2016).

*Citrus* species are among of the most widely cultivated and economically significant fruit crops (Xu et al., 2021). Given the large planting areas, citrus producers face multiple challenges relating to the dynamic environment and myriad stresses (Yu et al., 2020). Recent research showed that agricultural producers are facing several problems due to biotic and abiotic stresses like ubiquitous phytopathogens and changeable weather (high or low temperature, and soil salinity) which seriously reduce the Citrus yield and quality. For example, melanose disease caused by the fungal pathogen *Diaporthe citri*, which harms both leaves and fruits, contributes to massive reductions in yield and loss of quality (Mondal et al., 2007; Chaisiri et al., 2020), whereas citrus greening disease (Huanglongbing, HLB) is recognized as the most serious and fatal bacterial diseases threatening the citrus industry worldwide (Qiu et al., 2020; Yao et al., 2021). Unfortunately, HLB remains incurable, with all diseased plants eventually succumbing to the disease (Thapa et al., 2020). Currently, there are no known commercial citrus varieties with effective resistance to the phloem-residing HLB-associated bacterium *Candidatus* Liberibacter asiaticus (*C*Las; Iftikhar et al., 2016). Within a plant, the phloem is the predominant passageway for the long-distance transport of solutes and signaling, at the same time, provides an effective avenue of phloem-inhabiting bacteria spread systemically throughout a host plant (Welker et al., 2021). With respect to the breeding of resistant varieties, it is anticipated that on the basis comparative pathological, transcriptomic, and anatomical investigations using HLB-tolerant and -sensitive cultivars, phloem regeneration will become one of the most important and promising research directions in citrus production (Deng et al., 2019; Curtolo et al., 2020). It has long been established that KNAT6 (KNOX subfamily) is particularly enriched in phloem and required for correct lateral root formation in Arabidopsis (Dean et al., 2004). Similar findings have been reported in potato, in which the KNOX subfamily protein POTH1 interacts with the BEL subfamily protein StBEL5, a phloem-mobile messenger that regulates phloem transport activities (Mahajan et al., 2012; Hannapel et al., 2013). Thus, it would be of interest to investigate the potential function and underlying regulatory mechanisms of TALE family genes in host plant resistance to HLB pathogens.

To date, however, there has been no relevant research on the TALE family in sweet orange. Nevertheless, recent publication of the complete genome sequence of sweet orange now makes it feasible to conduct genome-wide identification and comparative analyses of the *TALE* gene family in sweet orange. In this study, we identified *CsTALE* genes in sweet orange, using which, we performed a comprehensive analysis, examining gene phylogeny, chromosomal position, duplication events, gene/protein structures, *cis*-acting regulatory elements (CREs), PPI networks, subcellular localization, and transcriptional activation, and undertaking yeast-two-hybrid validation. Moreover, we also examined expression profiles of all *CsTALE* genes in different sweet orange tissues and in response to different abiotic and biotic stresses. By adopting this integrative approach, we provide a basis for further elucidating the functional and mechanistic characteristics of the *TALE* genes. In addition, identification of stress resistance genes will provide a basis for effective engineering strategies to improve crop stress tolerance.

## Materials and Methods

### Identification and Phylogenetic Analysis

Publicly available information relating to the sweet orange genome sequences and gene annotations were downloaded from the National Center for Biotechnology Information (NCBI) and the *Citrus sinensis* Genome Annotation Project (Xu et al., 2013; Wu et al., 2018). All Hidden Markov Model (HMM) profile files of the TALE domain (Accession no. PF05920) were downloaded from the Pfam database, version 34.0^1^.

Sequences of *Arabidopsis thaliana* TALEs (*At*TALE), *Oryza sativa* TALEs (*Os*TALE), and *Populus trichocarpa* TALEs (*Pt*TALE) were obtained from previous studies (Hamant and Pautot, 2010; Zhao et al., 2019). Multiple alignments of TALE member amino acid sequences were performed using ClustalX software V2.1, employing default parameters with subsequent manual adjustment. A phylogenetic tree was generated using MEGA-X v10.2.4 software based on the neighbor-joining (NJ) algorithm, with the following parameters: Poisson correction, pair-wise deletion and bootstrap sampling (1000 replicates; random seed).

### Chromosomal Distribution of *CsTALE* Genes and Duplication Events

The chromosomal positions of *CsTALE* genes were extracted from the sweet orange genome annotation information in GFF3 format and visualized using Toolkit for Biologists stand-alone software v1.0986 (Chen et al., 2020). Chromosome size and gene density were determined with reference to the sweet orange genomic annotation information. *CsTALE* gene replication events were identified using a multiple collinear scan kit (MCScanX) program with default settings. For synteny analysis, the genome sequence and gene structure annotation files of sweet orange and Arabidopsis were inputted into One Step MCScanX, followed by the visualization with Dual Synteny Plot plugin embedded in TBtools software. KaKs_Calculator2.0 (MA model) was selected to calculate non-synonymous (Ka), synonymous (Ks), and Ka/Ks values.

### Gene Characteristic and Structural Analyses

The theoretical isoelectric point (pI) and molecular weight (MW) of entered protein sequence were estimated using Expert Protein Analysis System 3.0^2^ (Duvaud et al., 2021). The subcellular localization of CsTALE proteins was predicted using the online bioinformatics tools Plant-mPLoc^3^ and WoLF PSORT^4^. On the basis of the genome and coding sequences, the gene structure of each *TALE* gene was obtained using the Gene Structure Display server^5^. The conserved motifs of *Cs*TALE proteins were identified using the online MEME Suite Programs in classic mode. Domain-based analyses were performed using the SMART server^6^ in default mode (Letunic et al., 2021).

### *Cis*-Acting Regulatory Elements and Protein Interaction Network Predictions

In order to identify CREs in the promoter sequence of sweet orange *TALE* genes, we extracted genomic DNA sequences extending 2000 bp upstream of the transcription start site, and then submitted these to the PlantCare website^7^. Potential PPIs were predicted using the STRING online portal (Version 11.0^8^).

### Plant Materials and Treatments

The sweet orange materials used in tissue-specific expression pattern and stress response analyses were obtained from the National Center for Citrus Improvement, Hunan Agricultural University, Hunan Province, China. For analysis, three samples of different tissues (leaf, stem, and flower) were sampled from the same sweet orange plant at the flowering stage, and ripe fruits were subsequently obtained.

The different stress treatments performed in this study were carried out as previously described, with each experiment being conducted with three replicates (Song N. et al., 2021). For the purposes of stress analysis, we used 1-month-old sweet orange plants that had been grown in greenhouse at 25°C under an 8-h dark/16-h light photoperiod.

#### Salt Stress Assays

Sweet orange seedlings with good health and the same growth potential were transferred to flasks containing 100 mM NaCl, with sterile distilled water serving as a control. Samples then collected at 0, 12, 24, and 48 h after treatment.

#### Wounding Assays

The leaves of the well-growth sweet orange were gently stab a wound with a pipette tip, with non-wounded plants as a control. Samples were taken at 0, 12, 24, and 48 h after treatment.

#### High or Low Temperature Stress Assays

Sweet orange seedlings with good health and the same growth potential were transferred to plant growth cabinet at 40 or 4°C for high temperature and low temperature treatments, with normal growth conditions as a control. Samples were taken at 0, 12, 24, and 48 h after treatment.

For each stress type, three independent samples were harvested at 0, 12, 24, and 48 h after treatment, and then immediately snap-frozen in liquid nitrogen and thereafter maintained at –80°C until used for RNA extraction.

*Diaporthe citri* spores were incubated on oat agar medium at 25°C until germinating, A suspension of these spores (1 × 10^6^ spores/mL) was subsequently used to inoculate 1-month-old sweet orange plants using the spray method as previously described (Agostini et al., 2003). *C*Las inoculation was performed using to a slightly modified version of the method described by Martins Cristina de Paula Santos et al. (de Paula Santos Martins et al., 2015). *C*las-infected sweet oranges showing typical HLB symptoms were collected from commercial citrus growing plantations in the central south region of Hunan Province, China. The seedlings were graft-inoculated with budwood from *C*Las-free and *C*Las-infected sweet orange to obtain healthy and infected plants, respectively, as described previously (Suh et al., 2021). For all new mature leaves, the presence of *C*Las was examined based on qRT-PCR analysis for 6 months post inoculation (Maheshwari et al., 2021). Three independent samples were collected at 0, 24, and 48 h post inoculation and maintained as described above.

### RNA Extraction and Quantitative Real-Time PCR

Total RNA was extracted from sweet orange leaves using TransZol (TransGen Biotech, Beijing, China) in accordance with the manufacturer’s instructions. One microgram of total RNA was used for first-strand complementary DNA synthesis using a Goldenstar RT6 cDNA Synthesis Kit (Tsingke Biotechnology, Beijing, China). All qRT-PCR reactions were run and analyzed using a CFX96 Touch Deep Well Real-Time PCR Detection System (Bio-Rad, Munich, Germany) with a SYBR Green PCR Mastermix (Solarbio, Beijing, China). qRT-PCR was conducted following standard procedures and conditions as previously described (Peng et al., 2021b). qRT-PCR gene-specific primers were designed using Oligo7 software (Supplementary Table 7).

### Subcellular Localization

Amplified full-length *TALE* fragments were cloned into a linearized pCAMBIAI1132 vector between a CaMV35S promoter and green fluorescent protein tag using ClonExpress II One Step Cloning Kit (Vazyme, Nanjing, China). The resulting vectors were introduced into *Agrobacterium tumefaciens* EHA105 by electroporation, followed infiltration into *Nicotiana benthamiana* leaves using a needleless syringe. Subsequently, samples were viewed under a CarlZeiss LSM710 confocal laser scanning microscopy.

### Transcriptional Activation and Yeast Two-Hybrid Assay

The transcriptional activation of TALE was analyzed according to a previously reported method (Liu et al., 2021). The full-length sequences of TALE proteins were fused in a pGBKT7 vector. Subsequently, pGBKT7-TALE recombinant vectors and a negative control pGBKT7 empty vector were separately transformed into the yeast strain AH109 in accordance with the manufacturer’s protocol (Weidi Biotechnology, Shanghai, China). The resulting culture was diluted and dropped on SD/–Trp, SD/–Trp/–His/–Ade and SD/–Trp/–His/–Ade/X-α-Gal synthetic dropout medium (Clontech, Mountain View, CA, United States), followed by incubation at 28°C for 3 days.

In order to validate the interactions between members of the *CsTALE* gene family, we performed a yeast two-hybridization (Y2H) assay using the constructed bait (pGBKT7) and prey (pGADT7) vectors. Yeast transformation was performed using Yeastmaker Yeast Transformation System 2 (Takara, Tokyo, Japan) according to the manufacturer’s instructions. Recombinant vectors and negative (pGBKT7/pGADT7) and positive (pGBKT7-53/pGADT7-T) control vectors were separately transformed into yeast strain AH109 as described above. Thereafter, the transformed yeasts were plated on SD/–Trp/-His, SD/–Trp/–Leu/–His, and SD/–Trp/–Leu/–His/–Ade/X-α-Gal synthetic dropout medium and incubated at 28°C for 3 or 4 days.

## Results

### Genome-Wide Identification and Phylogenetic Analysis of *TALE* Genes in Sweet Orange

To identify TALE genes in sweet orange, initial candidates were retrieved from the NCBI and *Citrus sinensis* Genome Annotation Project databases. HMMER (Hidden Markov Model) matrices specific to TALE family were then constructed using HMMBUILD (HMMER-3.1) and scanned against the PFAM domain (PF05920). On the basis of a rigorous two-staged screening process, we identified a total of 18 TALE superfamily genes were identified in sweet orange, which account for approximately 0.06% of the entire sweet orange genome (29,445 predicted genes in sweet orange). These 18 TALE family members were named based on the order of their chromosomal location (*Cs*TALE1–*Cs*TALE18) and different transcripts were distinguished by the postscripts a/b/c/d. Lengths of the open reading frames of sweet orange TALE genes ranged from 582 to 2520 bp, and calculated theoretical MWs of the TALE proteins varied ranged 22.16–92.52 kDa. The gene id, protein sequence, physicochemical properties and subcellular localization prediction of the characterized TALE genes/proteins are presented in Supplementary Table 1. A pairwise identity (%) matrix revealed similarities among of the sweet orange TALE family nucleotide and amino acid sequences, among which, the highest degree of similarity (93.44/96.89%) was obtained for *CsTALE4* and *CsTALE16* (Supplementary Table 2).

**FIGURE 1:**
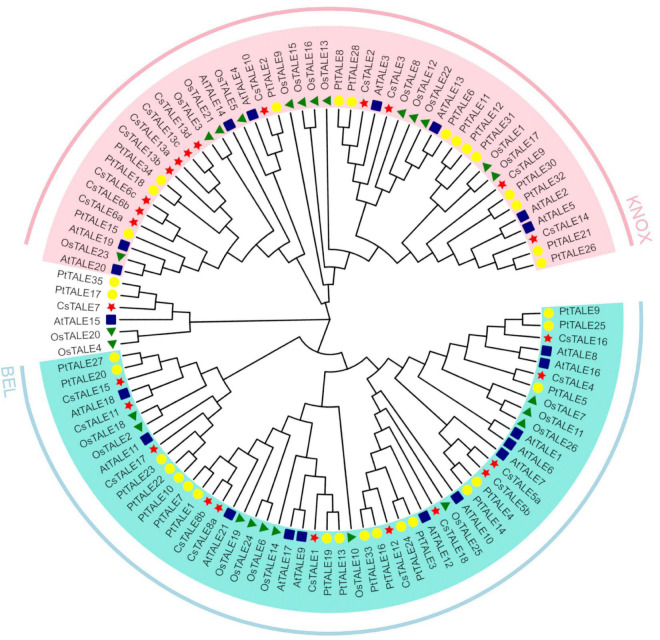
Phylogenetic tree of *Citrus sinensis*, *Arabidopsis thaliana*, *Oryza sativa*, and *Populus trichocarpa* TALE proteins. The phylogenetic tree was constructed from amino sequences using MEGA-X v10.2.4 software by the neighbor-joining method with 1000 bootstrap replicates. The TALE proteins are clustered into 2 subgroups, marked by different colors. Red star indicates *Citrus sinensis* TALEs (*Cs*TALE), blue square indicates *Arabidopsis thaliana* TALEs (*At*TALE), green triangle indicates *Oryza sativa* TALEs (*Os*TALE), and yellow circle indicates *Populus trichocarpa* TALEs (*Pt*TALE).

In order to reconstruct the evolutionary relationships among sweet orange and Arabidopsis TALE members, 39 aligned TALE protein sequences from sweet orange (18 TALE proteins), Arabidopsis (21 TALE proteins), rice (26 TALE proteins), poplar (35 TALE proteins) were used to generate a phylogenetic tree using MEGA X software and the neighbor-joining method (Figure 1). The phylogenetic distribution clearly indicated that the *TALE* genes clustered into two subfamilies (KNOX and BEL). The KNOX and BEL subfamilies were found to contain 10 and seven *CsTALE* genes, respectively, whereas *CsTALE7* forms a separate evolutionary branch. According to the current classification, these two clusters show obvious differences with respect to TALE sequence length, with the average length of KNOX and BEL subfamily proteins being 626 and 336 amino acids, respectively. The relevant grouping information, gene ids, and gene names are provided in Supplementary Table 3.

### Chromosomal Position and Duplication Analysis of *CsTALE* Genes

In order to determine the chromosomal distribution of *CsTALE* genes, the positions of *CsTALEs* were mapped on the chromosomes of sweet orange based on the NCBI *Citrus sinensis* genome sequence (Assembly Csi_valencia_1.0). The assessment results revealed a sparse distribution of the 18 *CsTALE* genes across all chromosomes, with the exception of chromosome 9 (Supplementary Figure 1), with variable *CsTALE* gene densities on individual chromosomes. The highest *CsTALE* gene frequency (three) was detected on chromosome 7, whereas chromosomes 1 and Un each harbored only a single gene (*CsTALE1* and *CsTALE18*, respectively). For the purposes of the present study, we defined tandem duplicated pairs as a genomic region harboring two or more neighboring *CsTALE* genes residing within a 20 kb sequence. Among all *CsTALE* genes, we detected only a single tandem duplicated pair (*CsTALE4/CsTALE5*) located adjacent to each other in a chromosomal region.

**FIGURE 2:**
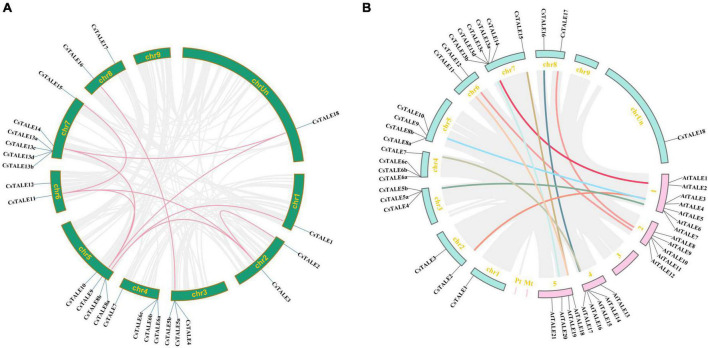
Duplication events of CsTALE genes. **(A)** Circle plot displayed duplication events of *CsTALE* genes based on the sweet orange genome. Colored lines indicated duplication of *CsTALE* genes. **(B)** The homologous gene pairs between sweet orange and Arabidopsis. Gray lines indicate all the collinear blocks within sweet orange and Arabidopsis. Other colored lines indicate TALE homologous gene pairs.

To further examine the relationship between genetic divergence and gene duplication, we performed comparative syntenic and duplication pair analyses. On the basis of our analysis of the sweet orange genome, we identified 10 segmental duplication events involving 10 *CsTALE* genes (Figure 2A). With the exceptions of chromosomes 4 and 9, segmental duplicates were detected on all chromosomes. *CsTALE3/9/11* were found to be involved in three duplication events, whereas others have been involved in two events (*CsTALE8/14/18*) or one event (*CsTALE1/4/15/16*). To assess the direction and strength of natural selection pressure, we estimated the rates of *K*a to *K*s substitution. The ratios of *K*a to *K*s for the 10 pairs of *CsTALE* genes were less than 1, ranging from 0.11 to 0.38, which indicates that the *CsTALE* gene pairs in sweet orange have undergone purifying selection during the course of evolution (Supplementary Table 4). *K*s values are routinely used to obtain approximate estimates of the evolutionary dates of segmental duplication events. We established that the duplication of *CsTALE* genes occurred during the from 3.70 Mya to 12.72 Mya, with a mean date of 8.86 Mya. In order to clarify the evolution and collinearity of sweet orange TALE family members among species, we sought to identify members of the *Cs*TALE family that had colinear relationships with those in the model plant *A. thaliana*, and accordingly identified 15 colinear gene pairs (Figure 2B and Supplementary Table 5). Syntenic relations of the TALE members among *C. sinensis*, *A. thaliana*, *O. sativa*, and *P. trichocarpa* are visualized in Supplementary Figure 2.

### Structural and *Cis*-Acting Regulatory Element Analysis of *CsTALE* Genes

**FIGURE 3:**
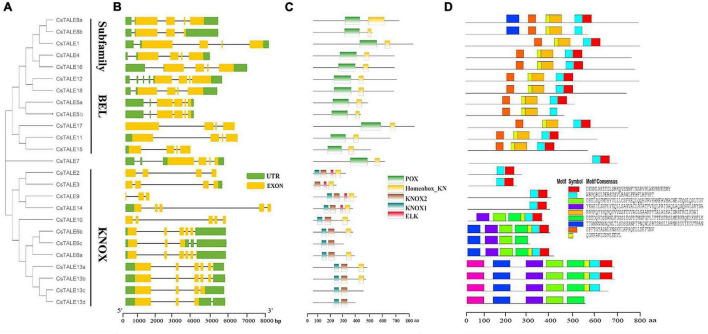
Phylogenetic relationships and gene/protein structure of CsTALE. **(A)** NJ phylogenetic tree was created in MEGAX software according to the CsTALE amino acid sequence. **(B)** Gene structure. Exons, UTR and introns are indicated by yellow rounded rectangles, green rounded rectangles and black lines, respectively. **(C)** Conserved domains. Different domains are represented in different color patches. **(D)** Protein motif. Schematic diagram of the conserved motifs of CsTALE proteins and the motif name indicated in the bottom right corner. The scale bar at the bottom is used to estimate the sizes of protein structure and gene structure.

Bioinformatics data obtained for proteins can enable us to establish correlations between structure and function, and in this regard, we determined motif/domain and exon/intron structures based on the corresponding amino acid and genome sequences (Figure 3). Structural analyses of these genes revealed that members of the BEL subfamily have the same number of exons, namely four, whereas KONX subfamily members are characterized by a diverse exon complement, ranging from three to six (Figure 3B). Moreover, all BEL subfamily members contain a 5′-UTR (except *CsTALE17*) and truncations to the 5′-UTR and/or 3′-UTR were found to be common among the *CsTALE* genes. In addition, BEL subfamily proteins all contain POX domains, whereas in the KONX subfamily, all proteins contain KONX1 and KONX2 domains, which are notably consistent with the subfamily clustering (Figure 3C). Some KONX subfamily proteins (*Cs*TALE2/3/9/14) are also characterized by an additional ELK domain. Conversely, with the exception of individual variants (*Cs*TALE6c/13c/13d), both BEL and KONX subfamily proteins possess a Homeobox_KN domain. In total, we identified 9 conserved motifs, designated motifs 1–9, in *Cs*TALE, with the number of conserved motifs in each *Cs*TALE ranging from 2 to 9 (Figure 3D). Notably, some characterized motifs were found to be present exclusively in one or the other subfamily, namely, motifs 5 and 8 in the BEL subfamily and motifs 3, 4, and 6 in the KONX subfamily.

*cis*-acting regulatory elements (CREs), located upstream of the promoter region, are essential sites for TFs that are associated with the initiation of transcription, and function as control centers for gene transcription. Among the CREs identified, abiotic stress responsive elements and phytohormone-related elements were selected for analysis. We detected marked differences in the number, location, and type of CREs among the promoters of different *CsTALE* genes (Supplementary Figure 3A), and also observed the presence of two or three-tandem CREs, some of which may overlap with others. Supplementary Figure 3B presents details of the analyzed CREs, including the total number of each CRE type and the corresponding CREs in each gene. Among these, most *CsTALE* genes contain all types, with CREs involved in abscisic acid response occurring at the highest frequency.

### Expression Profile of *CsTALE* Genes

Spatial patterns of gene expression can often provide valuable clues regarding gene function. Accordingly, to assess the potential functions of *CsTALE* genes in sweet orange development, we characterized the expression profiles of all 18 *CsTALE* genes in different tissues (stem, leaf, flower, and fruit) based on qRT-PCR analyses. Associated heat-maps revealed diverse patterns in the relative expression of *CsTALE* genes in different tissues (Supplementary Figure 4). In general, 11 *CsTALE* genes were found to be highly expressed in stems, whereas 14 show relatively low expression in fruit. *CsTALE13* is notably expressed at a high level in all examined tissues, and *CsTALE1/3/9/10* are highly expressed in stems, leaves, and flowers. In contrast, *CsTALE12/14* were observed to be weakly expressed in all tissues.

**FIGURE 4:**
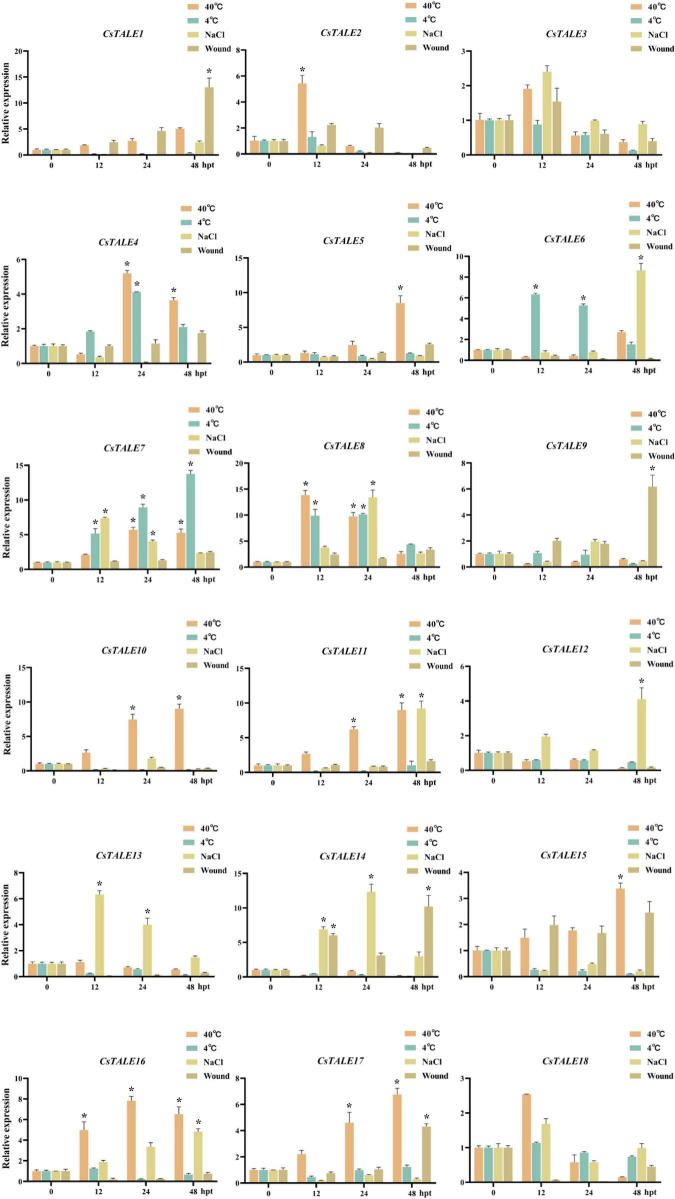
Expression levels of *CsTALE* genes under different abiotic stress treatment. The *Y*-axis represent the relative expression level of *CsTALE* genes and the *X*-axis indicate different time points post abiotic stress treatment. Different colors represent different stress treatment. The standard errors are plotted using vertical lines. * Represents significant difference (*p* < 0.05). The experiments in all panels were repeated three times with similar results.

To identify those *CsTALE* genes that play a potential role in stress responses, we exposed *Citrus* seedlings to bacterial and fungal infection (*C*Las and *D. citri*, respectively) and abiotic stresses (high and low temperature, salt, and wounding), and examined the expression patterns of the 18 *CsTALE* genes at 0, 12, 24, and 48 h post-treatment using qRT-PCR (Figure 4). qRT-PCR analyses revealed that most of the *CsTALE* genes underwent changes in expression in response to different stresses over the course of the experiment. For example, *CsTALE7/8* were found to be induced by salt and high and low temperature treatments, whereas *CsTALE11/16* were induced in response to both salt and high temperature, and *CsTALE1/17* were induced by wounding or high temperature treatment. Intriguingly, some *CsTALE* genes showed significantly contrasting expression patterns in response to different stress types. For example, whereas *CsTALE1/2/10/11/16/17* were induced by a high temperature, their expression was significantly inhibited by exposure to a low temperature treatment. In contrast, *CsTALE6* was characterized by the converse pattern of expression.

**FIGURE 5:**
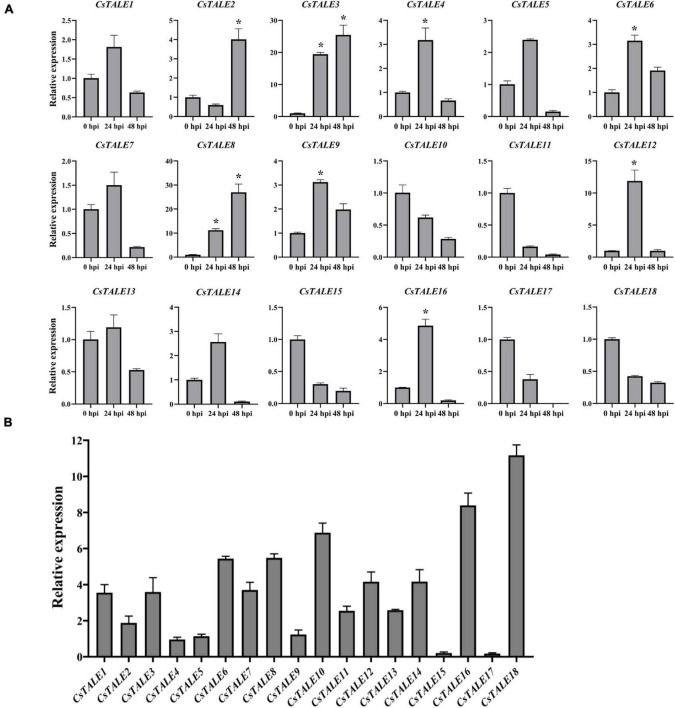
Expression levels of *CsTALE* genes under different biotic stress. **(A)** The *Y*-axis represent the relative expression level of *CsTALE* genes and the *X*-axis indicate different time points post *Diaporthe citri* inoculation. **(B)** The *X*-axis represented the different *CsTALE* genes and the *Y*-axis represent the relative expression level after *Candidatus* Liberibacter asiaticus-infected. The gene transcription levels in *C*Las-free plants were normalized as 1. The standard errors are plotted using vertical lines. * Represents significant difference (*p* < 0.05). The experiments in all panels were repeated three times with similar results.

We also investigated the expression of *CsTALE* in sweet orange infected with the fungal pathogen *D. citri* and bacterial pathogen *C*Las. Figure 5A shows the *CsTALE* genes differentially expressed in response to *D. citri* infection at 0, 24, and 48 h post-infection. Eight *CsTALE* genes were observed to be significantly up-regulated by *D. citri* inoculation, with greater than threefold changes, among which, *CsTALE4/6/9/12/16* showed highest up-regulated expression at 24 h, whereas the expression of *CsTALE2/3/8* peaked at 48 h. Conversely, the expression of four BEL subfamily genes (*CsTALE11/15/17/18*) and one KNOX subfamily gene (*CsTALE10*) was markedly inhibited. The expression profiles of *CsTALE* genes in *C*Las-infected sweet orange revealed that most of these genes were up-regulated in *C*Las-infected plants compared with healthy plants, although exceptions were noted. Specifically, we detected no appreciable changes in the relative expression of *CsTALE4*/*5*/*9*, whereas the expression of *CsTALE15*/*17* appeared to be strongly suppressed (Figure 5B).

### Subcellular Localization and Transcriptional Activation

**FIGURE 6:**
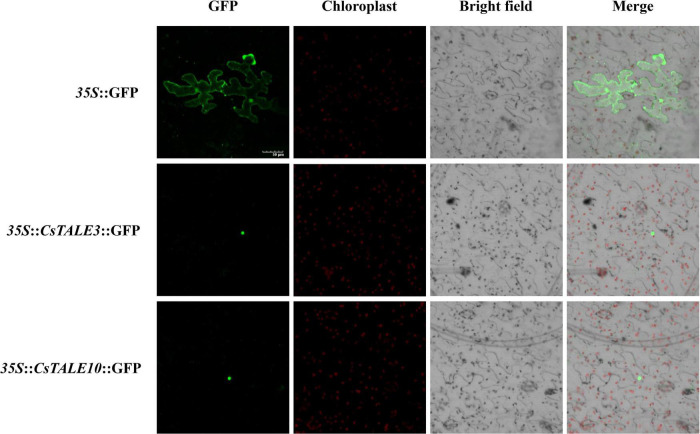
Subcellular localization of CsTALE. The fusion constructs (35S:TALE-GFP) and GFP (control) were separately transiently expressed in tobacco leaves and visualized under a confocal laser scanning microscopy. Bar denotes 50 μm.

To examine the subcellular localization of *Cs*TALE, we initially employed both the Plant-mPLoc and WoLF PSORT web-servers to predict subcellular localizations. Prediction results indicated that all these proteins are localized in the nucleus (Supplementary Table 1). To further substantiate these results, we generated 35S:TALE-GFP constructs and used these to observe transient expression in *N. benthamiana* leaves. This accordingly enabled us to confirm nuclear localization of the *Cs*TALE3/10 proteins (Figure 6).

**FIGURE 7:**
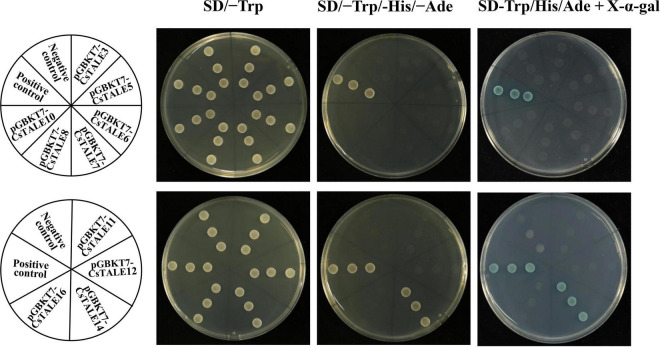
Transcriptional activation of CsTALEs. Transactivational analyses. Fusion constructs (pGBKT7-CsTALE) and negative control (pGBKT7 empty vectors), were transformed into yeast AH109 strain and incubated in SD/-Trp, SD/-Trp/–His/-Ade and SD-Trp/His/Ade + X-α-gal medium.

In order to ascertain whether these TALE proteins have transcriptional activation, we constructed yeast expression vectors (pGBKT7-TALE), which were used to transform the AH109 yeast strain. We accordingly found that all transformed yeasts grew normally on the SD/–Trp medium (Figure 7). pGBKT7-*Cs*TALE14 co-transformed yeast cells grew well on SD/–Trp/–Leu medium and turned blue on X-α-gal-supplemented SD–Trp/His/Ade medium, thereby indicating that they have transcriptional activation ability. However, yeast transformed with pGBKT7-*Cs*TALE3/5/6/7/8/10/11/12/16 and the negative control pGBKT7 empty vector were only able to grow on the SD/–Trp, and were neither able grow nor turned blue on the SD–Trp/His/Ade + X-α-gal medium. These observations thus tended to indicate that pGBKT7-*Cs*TALE3/5/6/7/8/10/11/12/16 did not show the transcriptional activation in transformed yeast.

### CsTALE Protein Interaction Network and Interaction Analysis

**FIGURE 8:**
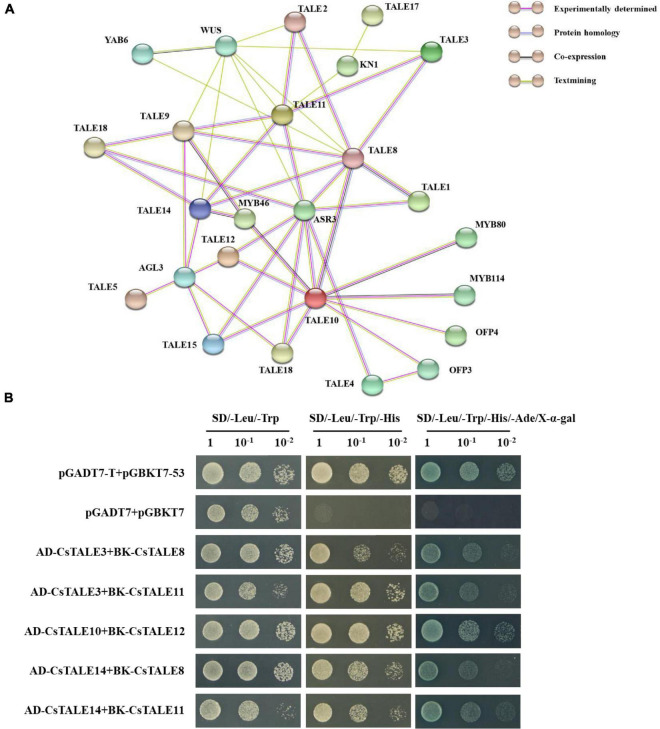
Interaction network and protein interaction of CsTALEs. **(A)** Interaction network of TALE. Nodes represent proteins, and lines represent protein interaction pairs. Line colors represent different types evidence of protein interaction pairs. **(B)** Yeast two-hybrid assays. The co-transformed yeast cells were diluted to different concentrations (1, 10^–1^, 10^–2^) and cultured in SD/–Leu/Trp, SD/–Leu/Trp/His and SD/–Leu/Trp/His/Ade + X-α-gal medium.

The web-based database for PPI networks, with predicted and known protein interactions, including direct (physical) and indirect (functional) associations, provides a valuable basis assessing the biological functions of uncharacterized proteins. The PPI network we constructed for *Cs*TALE proteins comprised 28 nodes and 49 edges, with an average node degree of 3.5 (Figure 8A and Supplementary Table 6). The network revealed that several *Cs*TALE proteins interact directly or indirectly with other *Cs*TALE members, among which *Cs*TALE8 and *Cs*TALE10 are predicted to interact with five and four *Cs*TALE proteins, respectively, and thus could represent the key connector proteins in the PPI network.

On the basis of this network, we performed Y2H assays to systematically assess the interactions between predicted pairwise *Cs*TALE proteins. These assays revealed that yeast co-transfected with *Cs*TALEs, including pGADT7-*Cs*TALE3/pGBKT7-*Cs*TALE8, pGADT7-CsTALE3/pGBKT7-CsTALE11, pGADT7- *Cs*TALE10/pGBKT7-CsTALE12, pGADT7-CsTALE14/pGBKT7 -*Cs*TALE8, and pGADT7-*Cs*TALE14/pGBKT7-*Cs*TALE11 complex vectors, can grow well on SD/-Trp/–Leu, and SD/–Trp/–Leu/–His media and colonies turned blue on SD/–Trp/–Leu/–His/–Ade medium supplemented with X-α-gal (Figure 8B). Moreover, the growth of all five recombinant yeast was comparable to that of the positive control and clearly distinct from negative controls (Supplementary Figure 5). These results unequivocally provide evidence to indicate the interaction between these pairs of *Cs*TALE proteins.

## Discussion

*TALE* family genes, which are widely distributed in both plant and animal genomes, play prominent roles in numerous cellular processes, growth, and stress responses (Wang et al., 2021). In recent years, benefiting from the notable advances in bioinformatics and genomic technologies, *TALE* family genes in *A. thaliana* (Bellaoui et al., 2001), *Solanum tuberosum* (Sharma et al., 2014), *Gossypium hirsutum* (Ma et al., 2019), and *G. max* (Wang et al., 2021) have been systematically studied and characterized. In contrast, there have been no comparable genome-wide studies and characterization of the *TALE* gene family in sweet orange. To rectify this deficiency, we performed a comprehensive integrative genomic analysis of *CsTALE* genes in sweet orange.

Different species have been found to vary considerably with respect to the number of TALE family members they harbor. In this study, we identified a total of 18 *CsTALE* family genes in sweet orange, which compares with the 35 in *Populus trichocarpa* (Zhao et al., 2019), 18 in *Ananas comosus* (Ali et al., 2019), and 14 in *Medicago truncatula* (Dolgikh et al., 2020), which could reflect differences in genome size and ploidy level. We established that the 18 *Cs*TALE proteins differ notably in terms of amino acid residues and physicochemical properties. In lines with expectations, as TFs, all identified *Cs*TALE members are predicted to be nuclear localized. Similar to the soybean and poplar TALEs, we found that the number of amino acid residues and MWs of KNOX subfamily *Cs*TALEs are considerably smaller than those in the BEL subfamily (Zhao et al., 2019; Wang et al., 2021).

In order to gain a better understanding of evolutionary relationships among the identified TALE proteins, we constructed a neighbor-joining phylogenetic tree, on the basis of which, the *Cs*TALE proteins were classified into two subfamily, BEL and KNOX, which is consistent with previously reported observations (Ruiz-Estévez et al., 2017). Notably, *Cs*TALE7 and *At*TALE15 were found to cluster in the branch of the phylogenetic tree separate from the other assessed TALES. Given that members of the same phylogenetic cluster are generally assumed have similar functions, we speculate that *Cs*TALE7 in sweet orange has a growth-related function comparable to that of *At*TALE15 (ATH1), which has been demonstrated to influence the growth of either vegetative or reproductive organs and represses stem development (Song et al., 2020). In addition, it has been established that the domains/motifs of BEL and KNOX subfamily proteins tend to show a strong subfamily specificity, which is also consistent with our classification results. Particular protein domains/motifs have been shown to contribute in defining the functionality of certain DNA binding and PPIs (Liu M. et al., 2019). For example, POX, a plant-specific domain found in BEL subfamily members, is reported to function in association with homeobox domains. In Arabidopsis, VAAMANA, a BEL1-like homeodomain protein, interacts specifically with KNAT6 and STM to promote appropriate inflorescence development (Bhatt et al., 2004). KNOX1 is known to exert potent effects in inhibiting the expression of downstream target genes, whereas KNOX2 has been found to be essential for homodimer formation, and a combination of both KNOX1 and KNOX 2 can form a MEINOX domain (Nagasaki et al., 2001). Furthermore, the ELK domain has been speculated to serve as a nuclear localization signal, as well as a PPI domain (Jia et al., 2020). Accordingly, we would anticipate the different domain/motif types of *Cs*TALE members might provide clues as to the distinct or specialized functions of these proteins, which thus should be examined by future studies. Gene structure analysis revealed that 13 and 11 of the 18 *CsTALE* genes contain 5’- and 3’-UTRs, respectively. Previous study has demonstrated that 5’-UTRs plays a role in regulating mRNA stability, whereas 3’-UTRs may function as miRNA binding sites, which would thus confer *CsTALE* genes with rich and complex properties with respect to the regulation of downstream genes (Peng et al., 2012). In summary, we detected similarities in protein/gene structures of *Cs*TALEs grouped within the same subfamily or clade, although structures typically differ between members of the different subfamilies. Hence, structural consistencies or discrepancies may also contribute to similarities or diversity in the function of *Cs*TALE members.

Common patterns of duplication events, including tandem, segmental, and genomic duplications, are among the most important factors influencing biological evolution and the expansion of different gene families in eukaryotic genomes (Peng et al., 2021a). Most *CsTALE* genes appear to have originated from segmental duplication, which has been established to be the main evolutionary driving force, followed by tandem duplication. By determining the ratios of *K*a to *K*s, we were able to characterize the evolutionary history and differentiation paths of *CsTALE* genes, which indicated that these genes have primarily evolved under the influence of purifying selection, and that KONX subfamily members appeared later than those in the BEL subfamily. These evolutionary patterns of *CsTALE* gene origin and divergence are similar to those reported for *G. max*, which thus tends to indicate that *TALE* gene families have evolutionarily conserved mechanisms and functions (Wang et al., 2021).

The correct identification of orthologous genes in extensively studied model plants may provide important clues as to the properties of newly discovered members (Kristensen et al., 2011). Accordingly, we sought to identify the biological functions of *CsTALE* genes based on comparative synteny analysis of these genes and *TALE* genes from the Arabidopsis genome. We detected a total of 15 collinear gene pairs between sweet orange and Arabidopsis, and can thus speculate that these paired genes may have originated from a single common ancestral gene and that their role may have been broadly conserved over the subsequent course of evolution. For example, *At*TALE21 (BEL1) and *At*TALE15 (ATH1) have been reported to play roles in complex networks involved in early developmental stages of the inflorescence meristem (Smith and Hake, 2003), and AtTALE3 (KNAT7) and AtTALE15 (BLH6) have been demonstrated to influence secondary cell wall development by specifically interacting with one another (Liu et al., 2015). In addition, several reports have described certain functionally enriched homologous genes, including AtTALE3 (STM; Cole et al., 2006), AtTALE17 (BLH2; Xu et al., 2020), and AtTALE20 (KNAT3; Pagnussat et al., 2007). Although numerous genetic resources provide valuable insight into the molecular bases of different gene functions, further investigations are required to define the biological functions and associated molecular mechanisms for each candidate gene. In this regard, our CRE analysis revealed the potentially diverse roles of the identified *CsTALE* genes implicated in regulation of different biological processes in sweet orange, including responses to different phytohormones and stress.

The findings of previous studies have indicated that the transcript abundances of TALE family members differ considerably among different tissues, in which they perform different biological functions (Wuddineh et al., 2016). Thus, TALE gene expression patterns can provide important information regarding the function of candidate genes. Our subcellular localization analysis based on gene expression profiles indicated that most *CsTALE* genes are expressed in the stem at a high level of expression, thereby indicating that these genes may have certain tissue-specific properties and fulfill different functions in different tissues. For example, potato POTH1, a KNOX family protein, has been shown interact with seven BEL family proteins based on Y2H screening, and thereby regulates shoot tip cytokinin levels and tuber formation (Chen et al., 2003).

Various environmental stresses, both abiotic and biotic, can have pronounced detrimental effects that contribute to substantial reductions in citrus crop yields and productivity (Sun et al., 2019). In this regard, the findings of a recent study provide evidence to indicate that the *Gh*BLH7/*Gh*OFP complex in cotton functions as a negative regulator in regulating resistance to Verticillium wilt by inhibiting lignin biosynthesis and the JA signaling pathway (Ma et al., 2020). Furthermore, on the basis of regulatory network analyses, *TALE* family genes have been predicted to be key factors mediating resistance to bacterial spot disease in pepper (Zhu et al., 2021). However, there have been comparatively few studies that have investigated the involvement of *CsTALE* genes in biotic stress responses, although we assume that their roles in this respect have been underestimated. Thus, in the present investigation, we utilized qRT-PCR analysis and integrated the overall levels of gene expression profiles to assess the magnitude of the responses of all identified *CsTALE* genes to different biotic and abiotic stresses. We accordingly observed that in response to *D. citri* infection, 13 of the 18 *CsTALE* genes were upregulated and five were down-regulated. Interestingly, in conjunction with PPI network analysis, we found that the expression profiles of *CsTALE* genes in response to *D. citri* infection indicate that for two *Cs*TALE proteins with predicted interactions, one is up-regulated and the other is down-regulated (e.g., CsTALE14–CsTALE18– CsTALE9–CsTALE11–CsTALE3 and CsTALE8–CsTALE10–CsTALE12). Moreover, when we examined the expression profiles of *CsTALE* genes in response to both *D. citri* and *C*Las infection, we found that *CsTALE3*/*6*/*8*/*12*/*16* were significantly upregulated and *CsTALE15*/*17* were strongly suppressed, thus indicating that these genes might play conserved roles in sweet orange disease resistance, via either positive or negative regulation. Moreover, *CsTALE10*/*11*/*18* were significantly upregulated in response to *C*Las infection, although were strongly inhibited by *D. citri* infection, which indicates that these genes may play unique immunological roles in HLB resistance. Given that *CsTALE* genes respond to different abiotic and biotic stresses to varying degrees, we speculate that these genes may play a dynamic regulatory role in the stress-induced gene regulation network of sweet orange; however, the underlying mechanisms need to be further investigated.

To gain further insights into the functions of *Cs*TALE proteins, we proceeded to investigate the subcellular localization and transcriptional activation of these proteins. Consistent with the established characteristic of TFs, we observed that *Cs*TALE3/10 localize exclusively to the nucleus. Subsequently, transcriptional activity experiments indicated that *Cs*TALE14 has transcriptional activation activity and may thus regulate the coordinate expressions of downstream genes. In contrast, *Cs*TALE3/5/6/7/8/10/11/12/16 showed no comparable transcriptional activation, which may indicate that these proteins initially need to form complexes with partners to exert their transcriptional activation function.

Protein–protein interactions network analysis revealed the identity of several functional partners among *Cs*TALE members. With respect to non-TALE interacting partners, OFP and MYB family proteins have been the most frequently reported TALE-interacting proteins (Gong et al., 2014; Wang et al., 2015), which is consistent with the interactions depicted in our PPI networks. In Arabidopsis, interaction between KNOX and BEL subfamily members has repeatedly been reported and demonstrated to play a key role in growth and developmental processes. The most well-studied and representative example of this phenomenon is the formation a transcriptional activation complex among BEL1 and KNAT1, KNAT2, STM, and KNAT5 proteins (Bellaoui et al., 2001). In this context, it is worth noting that the interaction of Arabidopsis protein pairs orthologous to *Cs*TALE14/*Cs*TALE11 and *Cs*TALE14/*Cs*TALE8 have previously demonstrated, and that the former pair has clearly characterized functions in the regulation of inflorescence development (Bhatt et al., 2004; Ragni et al., 2008). Somewhat surprisingly, in the present study, we identified interactions between the pairs CsTALE10/CsTALE12, CsTALE3/CsTALE8, and CsTALE3/CsTALE11, which have not previously been reported and could thus be species-specific.

Collectively, our characterization of the interactions of *Cs*TALE proteins reveals a certain degree of conservation, as indicated by comparisons with the homologous proteins in Arabidopsis. Nevertheless, we also identified certain differences indicative of multiple novel regulatory mechanisms among the *Cs*TALE family genes in sweet orange. A determination of complete or near complete interaction networks in further studies will hopefully enable us to clarify these mechanisms.

## Conclusion

In this study, we undertook a comprehensive and systematic analysis of the TALE family proteins in sweet orange. In total, 18 *CsTALE* genes were identified, which were unevenly distributed on nine chromosomes. We analyzed their phylogenetic relationships, duplication events, and protein/gene structures, and complemented these analyses with predictions of *cis*-acting regulatory elements and PPIs. In addition, we examined the expression of the 18 *CsTALE* genes in different tissues and in response to different abiotic and biotic stresses. Furthermore, yeast two-hybrid assays enabled us to determine the interaction between BEL and KNOX subfamily members. Taken together, the findings of this study yielded important new information that will provide a basis for further studies examining the roles of *CsTALE* genes in regulating sweet orange growth and stress tolerance, as well as contributing to future sweet orange breeding programs.

## Data Availability Statement

The original contributions presented in the study are included in the article/Supplementary Material, further inquiries can be directed to the corresponding author/s.

## Author Contributions

WP and YY performed the experiments, collected the data, and wrote the main manuscript text. JX and EP searched the literature and prepared the materials. SD and LD provided the value comments and analyzed the experimental data. TY and YW provided help in statistical and bioinformatics tools. DL and NS helped to typeset and proofread this manuscript. BW supervised the study, designed the experiments and assisted in editing the revisions of the manuscript. All authors contributed to the article and approved the submitted version.

## Conflict of Interest

The authors declare that the research was conducted in the absence of any commercial or financial relationships that could be construed as a potential conflict of interest.

## Publisher’s Note

All claims expressed in this article are solely those of the authors and do not necessarily represent those of their affiliated organizations, or those of the publisher, the editors and the reviewers. Any product that may be evaluated in this article, or claim that may be made by its manufacturer, is not guaranteed or endorsed by the publisher.

## Footnotes

1
https://pfam.xfam.org/

2
https://web.expasy.org/protparam/

3
http://www.csbio.sjtu.edu.cn/bioinf/plant-multi/

4
https://wolfpsort.hgc.jp/

5
http://gsds.cbi.pku.edu.cn/

6
http://smart.embl.de/

7
http://bioinformatics.psb.ugent.be/webtools/plantcare/html/

8
https://string-db.org/cgi/input.pl

## Supplementary Material

Supplementary Figure 1 Chromosomal location of *CsTALE* genes. Left bar represents chromosome length. The chromosome numbers are labeled on left of the chromosomes. Gene density was calculated based on the annotation information within a genomic region.

Supplementary Figure 2 Syntenic relations of the TALE members among *Citrus sinensis* and three representative plant species. Light-colored lines in the background represents the collinear relationship within *Citrus sinensis* and other plant genomes, and the deep red lines represent the collinearity of CsTALEs. Cs stands for *Citrus sinensis*, At for *Arabidopsis thaliana*, Os for *Oryza sativa* and Pt for *Populus trichocarpa*.

Supplementary Figure 3 Diagram of CREs in promoter sequences of *CsTALE* genes. **(A)** The box in different colors indicated different CREs. The description of the eight CREs were depicted on the right side. **(B)** The details of the CREs analysis statistics. Left bar chart showed the total number of each type CREs of the *CsTALE* genes. Upset plot showed the corresponding CREs of each gene. The black point indicated which sets are included in *CsTALE* genes.

Supplementary Figure 4 Quantitative real time-PCR analysis of *CsTALE* gene expression levels in various sweet orange tissues. Heatmap showing the expression of *CsTALE* genes in different tissues. qRT-PCR analysis of *CsTALE* genes expression in different tissues. The heat map was generated on the basis of log_2_ normalized intensity value. The color bar from blue-to-red indicated expression levels from high to low.

Supplementary Figure 5 The control of yeast two-hybrid analysis. pGBKT7-53/pGADT7-T were used as a positive control. TALE and the empty vector (pGADT7 or pGBKT7) were used as a negative control.
