# Supplementary figures and images for: TALE Transcription Factors in Sweet Orange (Citrus sinensis): Genome-Wide Identification, Characterization, and Expression in Response to Biotic and Abiotic Stresses
Source: Front Plant Sci. 2022 Jan 20;12:814252. doi: 10.3389/fpls.2021.814252 (PMC8811264; doi:10.3389/fpls.2021.814252)

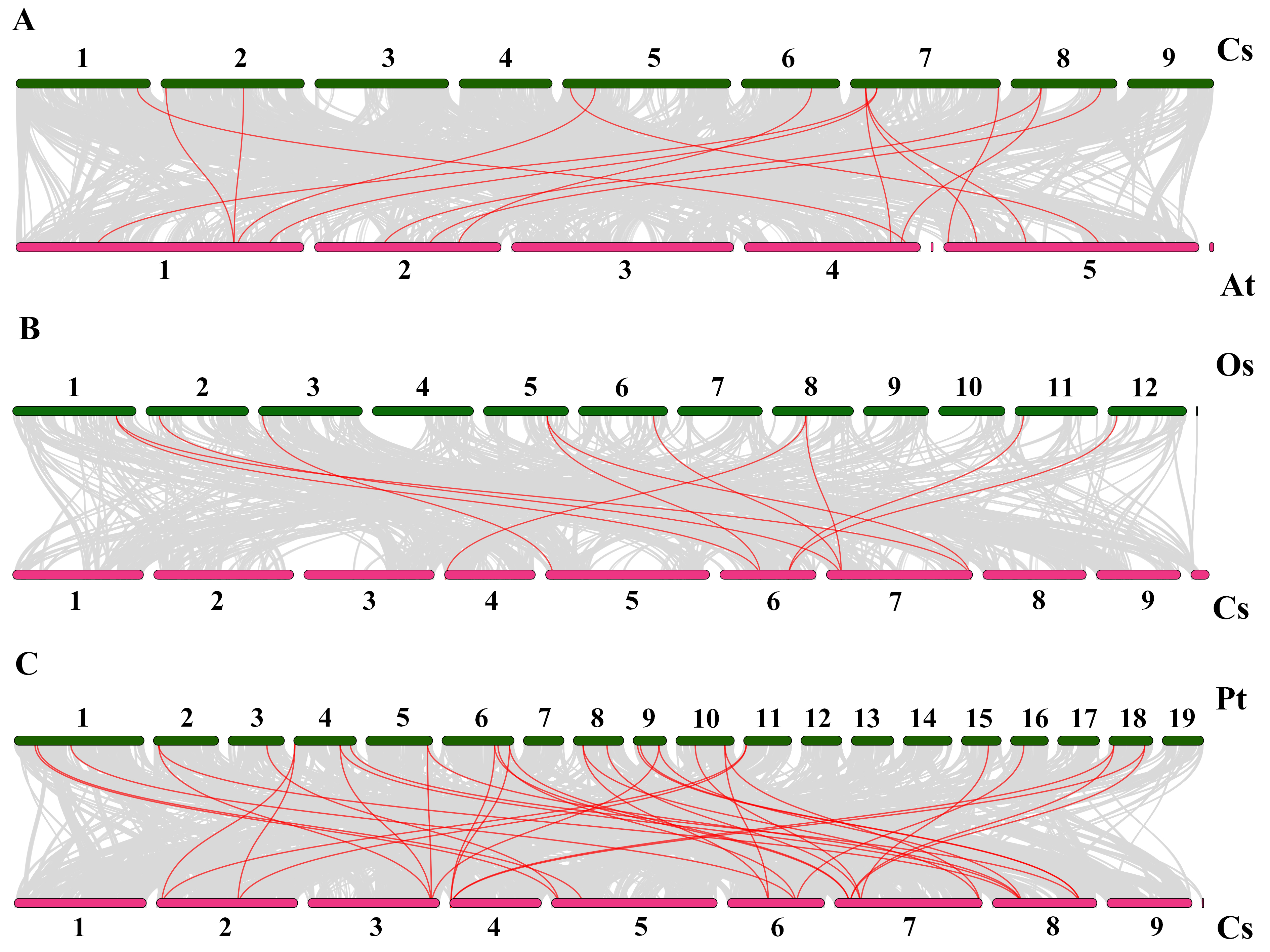

Supplement: Supplementary Figure 2 — Syntenic relations of the TALE members among Citrus sinensis and three representative plant species. Light-colored lines in the background represents the collinear relationship within Citrus sinensis and other plant genomes, and the deep red lines represent the collinearity of CsTALEs. Cs stands for Citrus sinensis, At for Arabidopsis thaliana, Os for Oryza sativa and Pt for Populus trichocarpa. [file Image_2.JPEG]

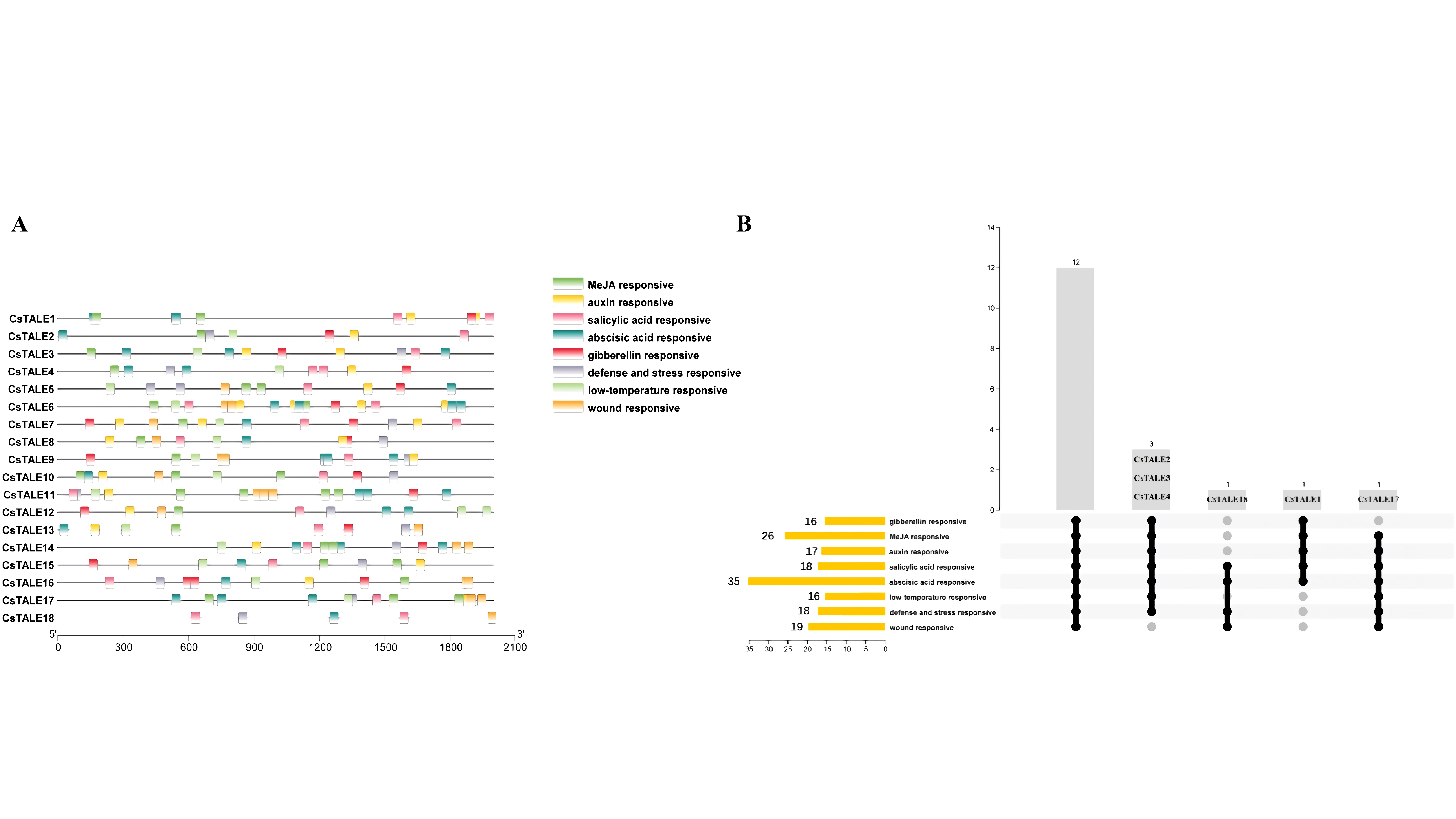

Supplement: Supplementary Figure 3 — Diagram of CREs in promoter sequences of CsTALE genes. (A) The box in different colors indicated different CREs. The description of the eight CREs were depicted on the right side. (B) The details of the CREs analysis statistics. Left bar chart showed the total number of each type CREs of the CsTALE genes. Upset plot showed the corresponding CREs of each gene. The black point indicated which sets are included in CsTALE genes. [file Image_3.JPEG]

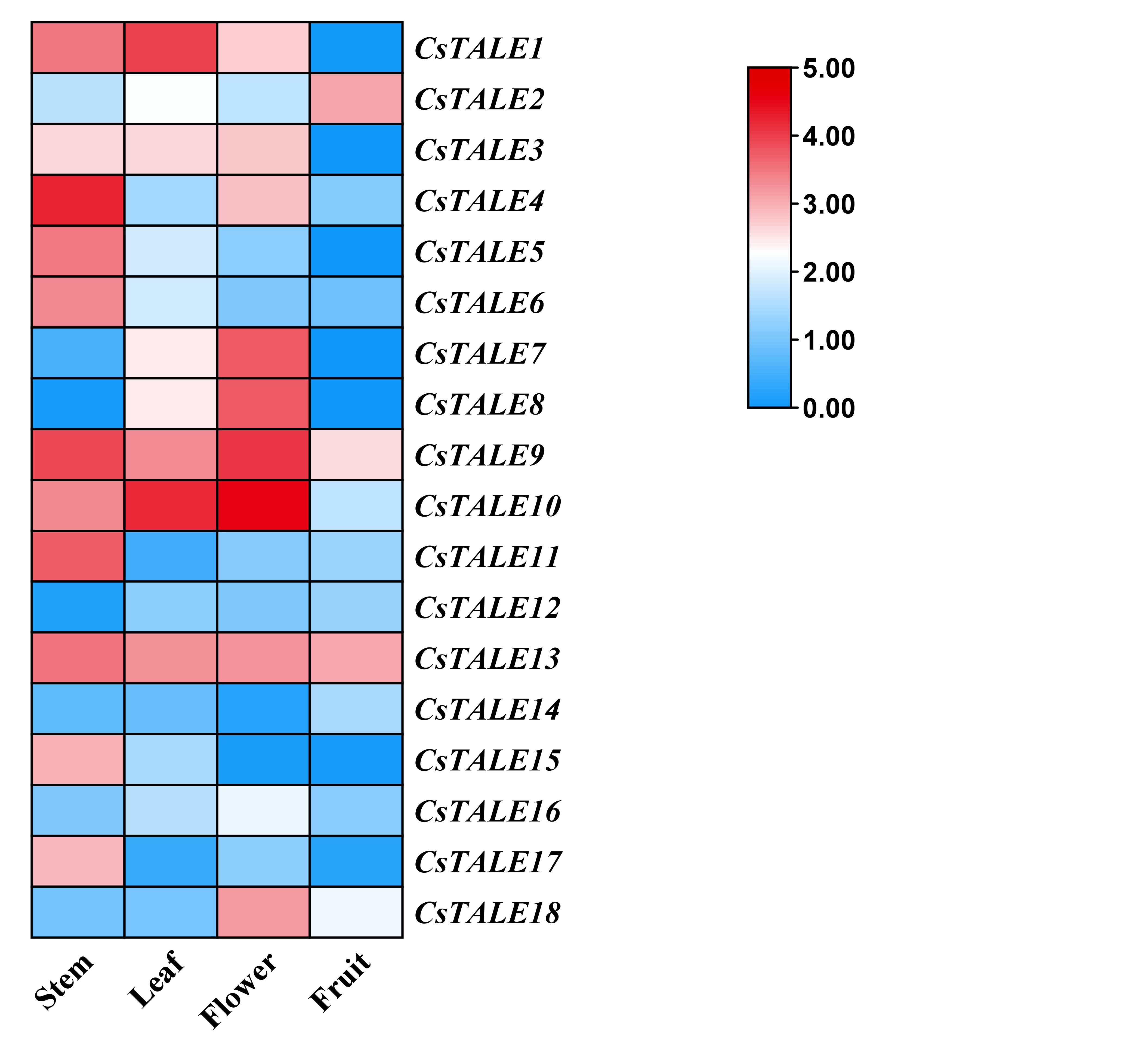

Supplement: Supplementary Figure 4 — Quantitative real time-PCR analysis of CsTALE gene expression levels in various sweet orange tissues. Heatmap showing the expression of CsTALE genes in different tissues. qRT-PCR analysis of CsTALE genes expression in different tissues. The heat map was generated on the basis of log2 normalized intensity value. The color bar from blue-to-red indicated expression levels from high to low. [file Image_4.JPEG]

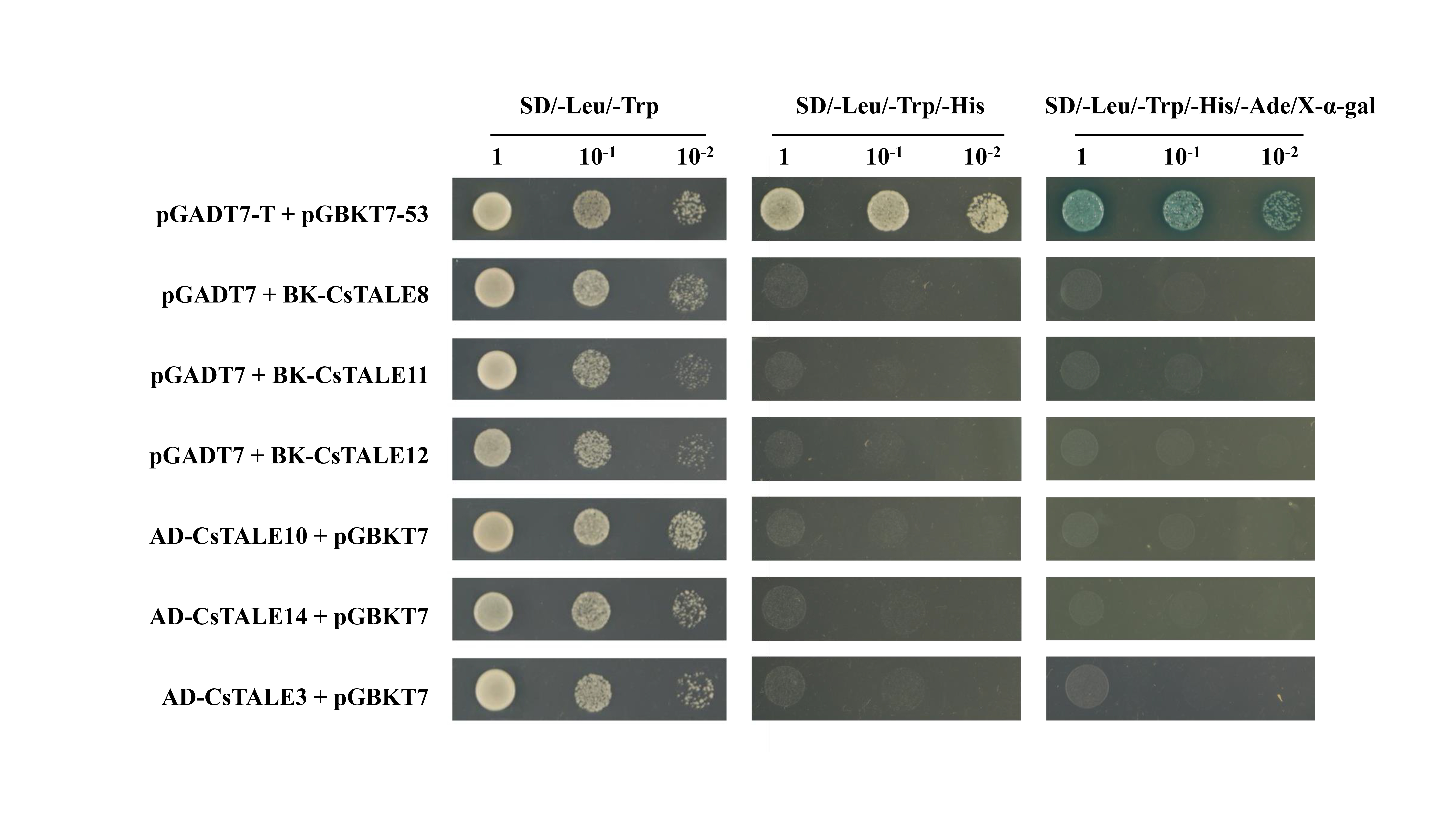

Supplement: Supplementary Figure 5 — The control of yeast two-hybrid analysis. pGBKT7-53/pGADT7-T were used as a positive control. TALE and the empty vector (pGADT7 or pGBKT7) were used as a negative control. [file Image_5.JPEG]
